# Range‐wide population genomic structure of the Karner blue butterfly, *Plebejus* (*Lycaeides*) *samuelis*

**DOI:** 10.1002/ece3.70044

**Published:** 2024-09-12

**Authors:** Jing Zhang, Aaron W. Aunins, Timothy L. King, Qian Cong, Jinhui Shen, Leina Song, Gregor W. Schuurman, Randy L. Knutson, Ralph Grundel, Jessica Hellmann, Nick V. Grishin

**Affiliations:** ^1^ Eugene McDermott Center for Human Growth and Development University of Texas Southwestern Medical Center Dallas Texas USA; ^2^ Department of Biophysics University of Texas Southwestern Medical Center Dallas Texas USA; ^3^ Harold C. Simmons Comprehensive Cancer Center University of Texas Southwestern Medical Center Dallas Texas USA; ^4^ U.S. Geological Survey, Eastern Ecological Science Center at the Leetown Research Laboratory Kearneysville West Virginia USA; ^5^ Department of Biochemistry University of Texas Southwestern Medical Center Dallas Texas USA; ^6^ U.S. National Park Service, Climate Change Response Program Fort Collins Colorado USA; ^7^ U.S. National Park Service, Indiana Dunes National Park Porter Indiana USA; ^8^ U.S. Geological Survey, Great Lakes Science Center Chesterton Indiana USA; ^9^ Department of Ecology, Evolution and Behavior, Institute on the Environment University of Minnesota Minneapolis Minnesota USA

**Keywords:** climate change vulnerability, genomic diversity, Karner blue butterfly, population genomics

## Abstract

The Karner blue butterfly, *Plebejus* (*Lycaeides*) *samuelis*, is an endangered North American climate change‐vulnerable species that has undergone substantial historical habitat loss and population decline. To better understand the species' genetic status and support Karner blue conservation, we sampled 116 individuals from 22 localities across the species' geographical range in Wisconsin (WI), Michigan (MI), Indiana (IN), and New York (NY). Using genomic analysis, we found that these samples were divided into three major geographic groups, NY, WI, and MI‐IN, with populations in WI and MI‐IN each further divided into three subgroups. A high level of inbreeding was revealed by inbreeding coefficients above 10% in almost all populations in our study. However, strong correlation between *F*_ST_ and geographical distance suggested that genetic divergence between populations increases with distance, such that introducing individuals from more distant populations may be a useful strategy for increasing population‐level diversity and preserving the species. We also found that Karner blue populations had lower genetic diversity than closely related species and had more alleles that were present only at low frequencies (<5%) in other species. Some of these alleles may negatively impact individual fitness and may have become prevalent in Karner blue populations due to inbreeding. Finally, analysis of these possibly deleterious alleles in the context of predicted three‐dimensional structures of proteins revealed potential molecular mechanisms behind population declines, providing insights for conservation. This rich new range‐wide understanding of the species' population genomic structure can contextualize past extirpations and help conserve and even enhance Karner blue genetic diversity.

## Graphical abstract

The Karner blue butterfly, *Plebejus* (*Lycaeides*) *samuelis*, is an endangered, climate change‐vulnerable species that has undergone substantial historical habitat loss and population decline. We performed a range‐wide assessment of genomic diversity and found that Karner blue populations had high levels of inbreeding and lower genetic diversity than closely related species. This new range‐wide understanding of the species' population genomic structure can contextualize past extirpations and help conserve and even enhance Karner blue genetic diversity.

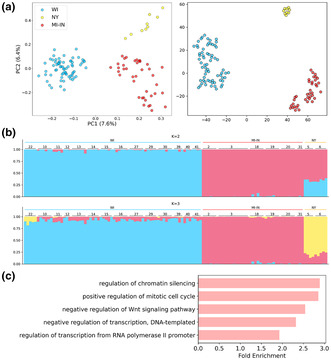

## 1 INTRODUCTION

The Karner blue butterfly, *Plebejus samuelis* (Nabokov, 1943; Talavera et al., 2013), is native to North America's Great Lakes region and the northeastern United States. The Karner blue's taxonomic designation is unsettled, and until recently it was classified as *Lycaeides melissa samuelis*. The genus was recently changed to *Plebejus* (Talavera et al., 2013) and the Karner blue has been proposed for promotion to full species (Forister et al., 2011). The current Integrated Taxonomic Information System (ITIS) designation for the Karner blue is *Plebejus melissa samuelis* (https://itis.gov/) but we follow the species‐level designation *P*. *samuelis* herein. The Karner blue has a wingspan of about 2.5 cm, and its dorsal wing surfaces are mostly blue in males but brownish blue with rows of orange crescents and black spots in females, and gray with orange and black dots beneath in both sexes. It is typically found in open, sunny habitats, particularly pine and oak savannas and barrens, but also sometimes occurs in roadsides, pastures, and old fields. Karner blues are bivoltine and their larval host plant is native wild lupine (*Lupinus perennis* L.; Grundel et al., 1998a; Grundel et al., 1998b).

Since the 19th century, the Karner blue has experienced a significant range‐wide population decline and contraction (Clough, 1992). This decline has been attributed to habitat loss and degradation, introduction of non‐native plant species that outcompete native wild lupine, pesticides, and other chemicals that harm Karner blues, and, recently, climate change (Lane, 1999; Maxwell, 1998; Patterson et al., 2020). The population decline precipitated listing of the Karner blue as an endangered species in Ontario, Canada, in 1990 (ECCC, 2023) and in the United States in 1992 (Clough, 1992), and the species is the focus of intensive recovery efforts (USFWS, 2019). Although the Karner blue once occurred in over a dozen states from Minnesota to Maine, as well as the Canadian Province of Ontario, remaining populations now mainly occur in Wisconsin, Michigan, New York, and New Hampshire (USFWS, 2019). Many of these populations continue to decline. For example, Karner blues from northwestern Indiana, which are included in this study, constituted a large population at the southern edge of the Karner blue range at the time of listing three decades ago (Schuurman et al., 2023). However, this population subsequently experienced a sustained, possibly climate change‐induced decline in the early 2000s. It was then driven to extirpation by a record‐setting warm year (2012) in which high early‐spring temperatures caused phenological mismatching between Karner blues and their larval hostplant, and hot, dry summer conditions caused early hostplant senescence (Patterson et al., 2020; Schuurman et al., 2023; USFWS, 2019).

The Karner blue has several close relatives in North America, including *P. melissa*, *P. idas*, *P. fridayi*, and *P. anna*. These morphologically similar species are distributed in different regions. *Plebejus fridayi* and *P. anna* co‐occur in California, with *P. fridayi* also found nearby in Nevada and the *P. anna* range extending northward across Oregon, Washington, and southern British Columbia. *Plebejus melissa* is widely distributed across the western United States and northern states in the central and eastern United States. *Plebejus idas* occurs further north across the extreme northern edge of the contiguous United States, as well as Canada and Alaska. These species also use different host plants. For example, *P. melissa* feeds on numerous legume genera including *Astragalus*, *Medicago*, *Glycyrrhiza*, and *Lupinus*—but not *L. perennis* (Scott, 1986; USFWS, 2019).

The Karner blue has been the subject of numerous genetic and genomic studies. In 2001, a panel of 14 microsatellite loci was characterized in the Karner blue, with four of these microsatellites being optimized for genotyping purposes (Anthony et al., 2001). However, population genetic results from using these loci have not been reported, perhaps because of the difficulty of using microsatellites to genotype Lepidoptera populations (Schmid et al., 2016; Sinama et al., 2011). Gompert et al. (2006) conducted the first population genetic analysis of the Karner blue and *P. melissa*, using 143 amplified fragment length polymorphisms (AFLPs) and partial sequences of mitochondrial *cox*1 and *cox*2 genes. The results supported treatment of the Karner blue as a distinct entity from *P*. *melissa*, but STRUCTURE (Pritchard et al., 2000) analyses did not identify strong differentiation between populations of the Karner blue east and west of Lake Michigan. Gompert et al. (2010) further sequenced the AFLP‐amplified regions of the Karner blue, *P. melissa* and *P. idas*, and found that the Karner blue differentiates strongly from other species. Forister et al. (2011) later addressed the same question using shotgun genomic data and agreed that the Karner blue should be treated as a distinct lineage and designated it as *Lycaeides samuelis*.

Resource limitation and climate change are known threats to Karner blues (Hoving et al., 2013; LeDee et al., 2013; Schuurman et al., 2023), but we have limited knowledge of how the response to these stressors may be geographically differentiated among populations (Grundel & Pavlovic, 2007; Li, 2020). Previous studies have suggested different populations of Karner blues are functionally divergent and may exhibit large‐scale differences in response to climate change (Hällfors et al., 2016). Understanding large‐scale genomic differences can be a first step in understanding the phylogeographic distribution of these functional differences and further understanding the nature of this species' adaptive capacity (Thurman et al., 2022). Given that the Karner blue continues to experience population extirpations and declines since listing—most notably in recent years along the southern periphery of the range in northern Indiana, northern Ohio, and southern Michigan (USFWS, 2019)—urgent questions are emerging about whether and how to conserve this species, including by possibly transferring individuals to new localities (Karasov‐Olson et al., 2021). Understanding the population genomic structure of Karner blue is critical for informing these decisions.

To facilitate restoration efforts and conservation planning for the Karner blue, we sequenced whole‐genome shotgun libraries of Karner blue samples from 22 localities to characterize this species' population structure and distribution of genomic diversity. We only sampled localities where the population was native (i.e., without a history of population augmentation or introduction from elsewhere^1^), and therefore excluded localities such as those in Ohio founded by individuals introduced from Michigan. We also sampled only extant populations (i.e., we did not sample museum specimens of extirpated populations), and thus did not include the Minnesota population, which became extirpated during or shortly before this study. Furthermore, we performed comparative genomic analyses among Karner blues and other species including *P. anna*, *P. fridayi*, *P. idas*, and *P. melissa*. We identified genomic features that might be associated with the reduced fitness of Karner blues, providing additional new insights for conservation.

## 2 MATERIALS AND METHODS

### 2.1 Sample collection, library construction, and sequencing

Karner blue butterflies were non‐lethally sampled for genetic analysis from four states in 2010, 2011, and 2012 following the general procedure of hind wing sampling in Hamm et al. (2010; Appendix S1). All samples were collected by personnel with training in the handling of the Karner blue and under USFWS permits. Samples consisted of wing clips (approximately 3 mm^2^ in surface area) from the hind wing with an effort to include part of the anal vein, which were immediately placed in 180 μL of cell lysis buffer (Qiagen Cat. No. 158116). Samples were shipped on ice to the USGS Eastern Ecological Science Center at the Leetown Research Laboratory (USGS‐EESC‐LSC), Kearneysville, WV. Samples were stored at −20°C or −80°C until DNA extraction. Genomic DNA was extracted using Qiagen Puregene (Qiagen Cat. No. 158023), Qiagen DNEasy (Qiagen Cat. No. 69504), or Macherey‐Nagel NucleoSpin Tissue (Macherey‐Nagel Cat. No. 740952.50) kits following the manufacturer's instructions. DNA was quantified using a Qubit dsDNA HS Assay kit (ThermoFisher Cat. No. Q32851). Pair‐end libraries were then prepared using NEBNext Ultra II FS DNA Library Prep kit (New England Biolabs Cat. No. E7805L) and sequenced on an Illumina HiSeq X10 system. Genomic data of other previously sequenced species, including *P. anna*, *P. fridayi*, *P. idas*, and *P. melissa*, were included in this study for comparison to the Karner blue (Appendix S2), most of which were included in Zhang et al. (2023). An additional *P. melissa* was sequenced in this study using different methodology from Zhang et al. (2023) and is described in the next section with the goal of obtaining a more complete‐genome assembly. All Karner blue sequence reads as well as those from other species can be retrieved from NCBI BioProject PRJNA995790 and corresponding BioSample accessions in Appendices S1 and S2.

### 2.2 Assembly and annotation of the *P. melissa* genome

DNA was extracted from a female *P. melissa* specimen collected from Laramie County, Wyoming, in 2021 (NCBI BioSample SAMN37522558), using the NEB Monarch HMW DNA Extraction kit (NEB Cat. No. T3060L), following the manufacturer's protocol. An Oxford Nanopore library was prepared using the LSK110 Ligation kit (Oxford Nanopore Cat. No. SQK‐LSK110), with two modifications: fragmenting the long genomic DNA using 29G needles and introducing a size selection step before adapter ligation. The concentration of the library was measured using Qubit, and around 20 fmol was loaded onto a flow cell R9.4.1. We performed base calling using Guppy and assembled draft genomes using NextDenovo (https://github.com/Nextomics/NextDenovo) and NECAT (Chen, Nie, et al., 2021), respectively, with the default settings. We further integrated the two assemblies by quickmerge (Solares et al., 2018), removed redundant contigs by Purge Haplotigs (Roach et al., 2018), and corrected errors in the assembly by Inspector with the default settings (Chen, Zhang, et al., 2021). We used the homology‐based annotation method, GeMoMa (Keilwagen et al., 2019) with the default settings, along with the annotation of the *P. argus* draft genome to annotate the draft assembly of *P. melissa*. Visualization of genome quality was generated by https://github.com/ammaraziz/assembly‐stats. Meanwhile, we predicted function of proteins encoded in the *P. melissa* genome by mapping these proteins to FlyBase (Gramates et al., 2022) with BLASTP (Altschul et al., 1997). Due to high conservation of the Z‐chromosome in Lepidoptera (Fraisse et al., 2017), we identified possible Z‐linked scaffolds by mapping *Heliconius* Z‐linked proteins to our draft genome using TBLASTN (Altschul et al., 1997). The draft *P. melissa* genome is deposited in NCBI with accession number JBBPCG000000000.

### 2.3 Assembling sequences of Karner blues and SNP callings

We performed SNP calling of Karner blue samples (Appendix S1) following our established pipeline described in Cong et al. (2021). Briefly, the adapters and low‐quality portion of the sequencing reads were trimmed using Trimmomatic‐0.36 (Bolger et al., 2014), and overlapping read pairs were merged using PEAR and the default settings (Zhang et al., 2014). The resulting reads were mapped to the *P. melissa* assembly using BWA‐MEM (Li, 2013), and we kept the reads that were mapped unambiguously in the correct orientation. Using the BWA‐MEM results, we computed the total sequencing depth in each 100 bp window. We considered windows with too high or too low total depth to be less confident. For example, windows with high depth might cover repetitive regions, whereas windows with very low depth may be misassembled or highly variable. Therefore, we only used the segments that contain at least three consecutive windows (≥300 bp) with depth between 0.25 and 2.5 times the median sequencing depth, and reads mapped to genomic regions other than these regions were discarded. In total, we obtained 331,230,775 “good positions,” around 79% of assembly size of *P. melissa*, our reference. We performed SNP calling with samtools (Li et al., 2009) for each specimen using alignments between reference and reads after the two cleaning‐up protocols.

### 2.4 Assembling sequences of closely related species and calling SNPs

The same SNP calling protocol described above for the Karner blue samples was employed for closely related species (Appendix S2). However, because many of these specimens were from museums and their DNA could be contaminated and highly degraded, we developed and sequentially applied the following two protocols (A and B) to clean up the alignments.

#### 2.4.1 Protocol A

For each 30 bp sliding window applied to the alignment between the reference genome and the reads, we clustered all the reads into groups of similar sequences using the following procedure. We ranked reads based on their sequence identity to the query from high to low. The first read initiated a cluster. Starting from the second read, each new read was compared to the first sequence of each cluster and assigned to the first cluster whose first sequence had no more than one mismatch from the current sequence. If a new read could not be assigned to existing clusters, a new cluster was initiated with this read as the first member. For each cluster, we computed its size and the average number of mismatches to the query and we considered a cluster to be good if its size was at least half of the largest cluster size and the number of mismatches was no larger than the minimal mismatches for all clusters +2. If the number of good clusters was no more than 2, we marked the reads that were not in the good clusters as bad reads; otherwise, we marked all the reads as bad. All the bad reads were discarded.

#### 2.4.2 Protocol B

For each read (a target), we obtained other reads that overlapped with it in the genomic regions they mapped to and counted the number of reads that were consistent and inconsistent with it. We considered a read to be consistent if it overlapped with the target read for at least 20 bp and the sequence identity was above 95%; otherwise, we considered a read to be inconsistent if it had at least two mismatches from the target and sequence identify <95%. If the number of consistent reads for a target was more than twice the number of inconsistent reads, the target read was kept. Otherwise, the target read was discarded.

We performed SNP calling with samtools (Li et al., 2009) for each specimen using alignments between reference and reads after the two cleaning‐up protocols. The final SNP genotype data are accessible from U.S. Geological Survey Science Base at https://doi.org/10.5066/P143CSPQ.

### 2.5 Analysis of population structure in the Karner blue

Due to the small sample size of Karner blues from each regional collection (range of 3–6 samples per locality) but large number of loci, we applied Reich's *F*_ST_ estimator (Reich et al., 2009) on localities with four or more individuals sampled. The geographic distance between localities was calculated using the Python package haversine (https://github.com/ajepe/haversine). A Mantel test was performed to test for isolation by distance using the Python script mantel.py from sci‐kit bio (https://github/scikit‐bio) with *F*_ST_ standardized as *F*_ST_/(1 − *F*_ST_).

We used BA3‐SNPs (Mussmann et al., 2019) to calculate the inbreeding coefficients of the Karner blue samples at each locality. BA3‐SNPs requires more than one allele at each locus and loci are assumed to be in linkage equilibrium, but deviations from Hardy–Weinberg equilibrium are allowable. Loci were selected with the following criteria: (1) at least two samples were present for each locality at each selected locus; (2) each locus had at least two different alleles, and (3) any two loci were more than 50 kb apart. Two runs of BA3‐SNPs were started to ensure convergence. The final input comprises 6863 loci and 116 individuals across 22 localities. We initiated two BA3‐SNPs runs, each with a distinct seed, executing 12 million iterations with a 5 million iteration burn‐in phase. The mixing parameters for allele frequencies (‐a) and inbreeding coefficients (‐f) were set at .5 and .06, respectively. This configuration was selected to achieve a suitable acceptance rate (ranging from 20% to 65%) and to ensure effective mixing. The convergence of two runs was checked by Tracer 1.7 (Rambaut et al., 2018).

Principal component analysis (PCA) with smartpca in Eigensoft (Patterson et al., 2006) was performed with bi‐allelic loci that met the following criteria: (1) more than 75% of individual samples in the analysis were covered with a sequence depth above 2, and (2) both alleles were present in more than four samples. To investigate if additional principal components would reveal more subtle population structure, we used t‐SNE (Van der Maaten & Hinton, 2008), a dimension reduction technique, to summarize the information from the first 10 principal components. Additionally, we used STRUCTURE (Pritchard et al., 2000) to investigate population structure using loci that met the following criteria: (1) more than 50% of samples were covered at each locus with a sequence of depth above 2, and (2) both alleles were present in more than four samples. Unlike PCA, which calculates and decomposes the covariance between samples, STRUCTURE places individuals into clusters that maximize conformance to Hardy–Weinberg equilibrium and minimize linkage disequilibrium. We probed various population numbers (*K*) ranging from 1 to 7 in our STRUCTURE analysis with the ADMIXTURE model, applying 10 replicates for each *K*. Each replicate involved a burn‐in of 100,000 iterations, followed by 100,000 post‐burn‐in iterations, with a sampling interval of 100 iterations. Various methods such as likelihood, Evanno method implemented in StructureHarvester (Earl & vonHoldt, 2012), and MedMeaK/MaxMeaK (Puechmaille, 2016) were used to analyze STRUCTURE results (see Appendices S3–S5). After obtaining the major clusters, we looked for additional population structure within each major cluster through hierarchical structure analyses (Vähä et al., 2007). For each major cluster identified, we repeated the above analysis. For the MI‐IN population, we probed *K*s ranging from 1 to 7, while for WI population, we probed *K*s ranging from 1 to 10. CLUMPAK was used to summarize the replicate results across *K*s (Appendices S3–S5 and Figures S1–S3).

To identify genes that may contribute to the differences among major groups of Karner blues, we calculated the percent divergence of proteins between each pair of groups. For each pairwise comparison, we only considered amino acid positions that were present in more than 40% of samples in both groups, which we refer to as “good positions,” and we only considered proteins with more than 200 “good positions.” The “good positions” did not have to be contiguous in one stretch. The following criteria were used to identify diverged positions among “good positions” and determine protein divergence: (1) the most frequent amino acid occurred in more than 70% of samples in both groups, and (2) the most frequent amino acids in the two groups were different. The divergence was calculated as the ratio of these “divergent positions” to the total number of “good positions.” We obtained a set of proteins with divergence higher than average in all three pairwise comparisons and performed gene ontology (GO) term enrichment analysis (geneontology.org). The enriched GO terms were identified by binomial tests using all proteins with at least 200 “good positions” as the background.

### 2.6 Genomic comparison of the Karner blue with closely related species

We compared the genome‐wide diversity of the Karner blues collected in this study against samples of *P. fridayi*, *P. anna*, *P. idas*, and *P. melissa* (Appendix S2). We sequenced fewer samples for other species—for example, we have only 13 samples of *P. fridayi*. Because we were concerned that differences in sample size may bias our analysis, that is, rare alleles in a species will only be revealed when the sample size is large, we randomly selected 13 samples from each species for comparisons. We chose a different set of random samples for each gene to avoid biases due to sample selection. We excluded nucleotide positions that were covered by fewer than two samples and counted the number of positions with at least two alleles, and each allele present in at least two samples. We calculated the ratio of such multiallelic loci per 10 kb window and compared such ratios across different species. Meanwhile, we took into account the disparity in collection years of Karner blue specimens, gathered in 2010 and 2011, and other closely related species collected over a broad time range, dating as far back as 1899 and as recent as 2021. This temporal variation in sample collection presented a risk of introducing biases, potentially leading to an artificially inflated perception of genetic diversity in species other than Karner blue. To rigorously verify the genetic diversity and mitigate these biases, we implemented a specific strategy in our pair‐wise *t*‐test. This involved, for each pair of *P. fridayi* or *P. melissa* specimens, calculating the differences in collection years and geographical distances. We then selected a pair of Karner blue specimens, ensuring that the year difference was at least as large and the geographical distance was equal to or up to twice the amount of the compared pair. We computed the ratio of loci with at least two alleles for both pairs and repeated this process across all *P. fridayi* or *P. melissa* pairs. The final step entailed comparing these ratios between the *P. friday*i or *P. melissa* pairs and the Karner blue pairs using a paired *t*‐test, employing “scipy.stats.ttest_rel” with the alternative hypothesis of “greater.” In addition, we also calculated the ratio of bi‐allelic loci for positions with depth of more than 4 (or 8) in a single sample of *P. fridayi*, *P. melissa*, and Karner blue.

Principal component analysis (PCA) with smartpca in Eigensoft (Patterson et al., 2006) was performed with bi‐allelic loci that met the following criteria: (1) more than 75% of individual samples in the analysis were covered with a sequence depth above 2, and (2) both alleles were present in more than four samples. To investigate if additional principal components would reveal more subtle population structure, we used t‐SNE (Van der Maaten & Hinton, 2008), a dimension reduction technique, to summarize the information from the first 10 principal components. Because genes linked to the Z‐chromosome exhibit higher levels of divergence than autosomes in previous studies examining evolutionary relationships among species of closely related butterflies in North America (Cong et al., 2019; Nelson et al., 2021), PCA and t‐SNE also included Z‐linked genes examined separately for comparison to autosomal genes.

In an attempt to identify genes associated with low diversity in the Karner samples relative to other closely related species, we identified 10 kb windows with significantly lower divergence (*p* < .01 using binomial tests) in the Karner blue but higher divergence (*p* < .01 by binomial tests) in both *P. fridayi* and *P. melissa*. We term such windows as “loss of diversity” regions and further identify genes that overlap with them. We extended the range of each protein‐coding gene 2000 bp upstream and downstream to account for the regulatory regions. We considered a gene to reside in the “loss of diversity” regions of the Karner blue if its range overlapped with such regions (10 kb windows) by ≥70%. We performed GO term enrichment analysis of such genes to identify biological processes affected by loss of diversity.

### 2.7 Identification of candidate SNPs affecting protein structure in the Karner blue

To identify amino acid positions with variations that may affect protein stability or functions in the Karner blue, we used the following criteria. First, a position was covered by more than 50% of the Karner blue specimens and more than 5, or more than 20% (whichever is larger) specimens in each of the other species. Second, if 95% of Karner blue specimens had the same amino acid at a position, we defined that as the “prevalent allele”. Third, the “prevalent allele” of the Karner blue is different from the dominating (present in ≥80% samples) amino acid of each closely related species. Fourth, the prevalent allele of the Karner blue is present in ≤5% of specimens in each closely related species. We modeled the structures of proteins containing such positions and with lengths <1500 amino acids by OmegaFold (Wu et al., 2022) and calculated the solvent accessibility area of the residue by mkdssp (Kabsch & Sander, 1983). We considered the residues with relative accessibility above 25% to be exposed on the protein surface.

## 3 RESULTS

### 3.1 *Plebejus melissa* reference assembly and annotation

**FIGURE 1:**
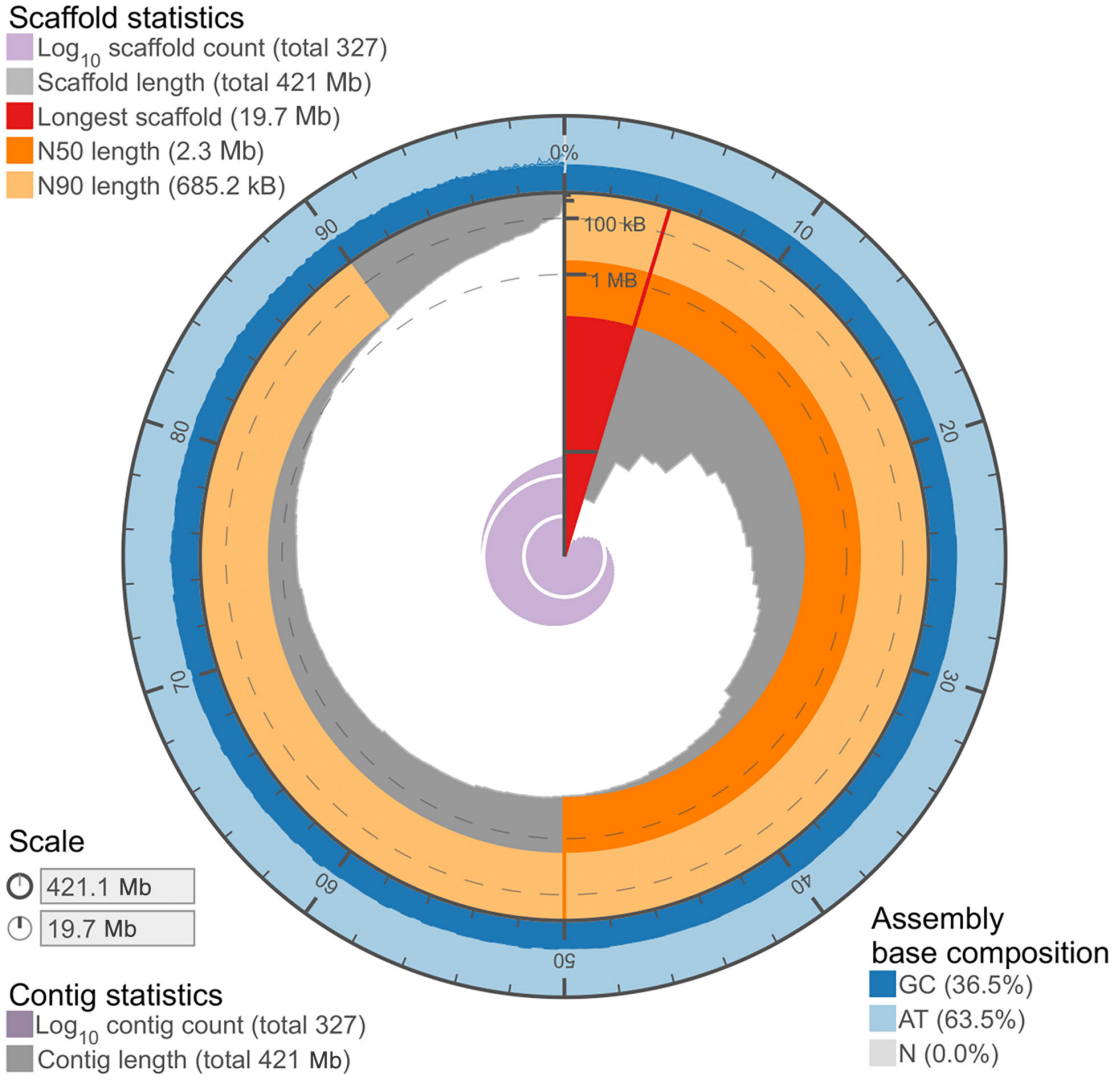
Statistics of *Plebejus melissa* assembly.

We obtained 14.5 Gb of data containing 5.5 million reads with an N50 of 15.2 kb from Oxford Nanopore Technology for *P. melissa*, after adapter trimming and removal of low‐quality base pairs using Guppy. This resulted in an estimated genome coverage of around 35× for *P. melissa*, whose genome is estimated to be 440 Mb in size based on flow cytometry. Two genome assemblies were generated using NextDenovo and NECAT assemblers, with initial N50 values of 874 and 514 kb, respectively. However, after applying quickmerge, the genome continuity was improved by a factor of 3, resulting in an N50 of 2.2 Mb (Figure 1). After removing redundant scaffolds with Purge Haplotigs and assembly correction with Inspector, the final polished genome consists of approximately 420 Mb spreading across 327 contigs with an N50 of 2.3 Mb. The completeness of this draft genome is 95% based on the presence of genes encoding BUSCO (Manni et al., 2021) Eukaryota conserved proteins in the genome. We annotated 12,914 protein‐coding genes in this reference genome by GeMoMa, and the predicted function for 9207 of them based on homology to Flybase entries (Appendix S6).

### 3.2 Genome resequencing and clustering of Karner blue samples

**FIGURE 2:**
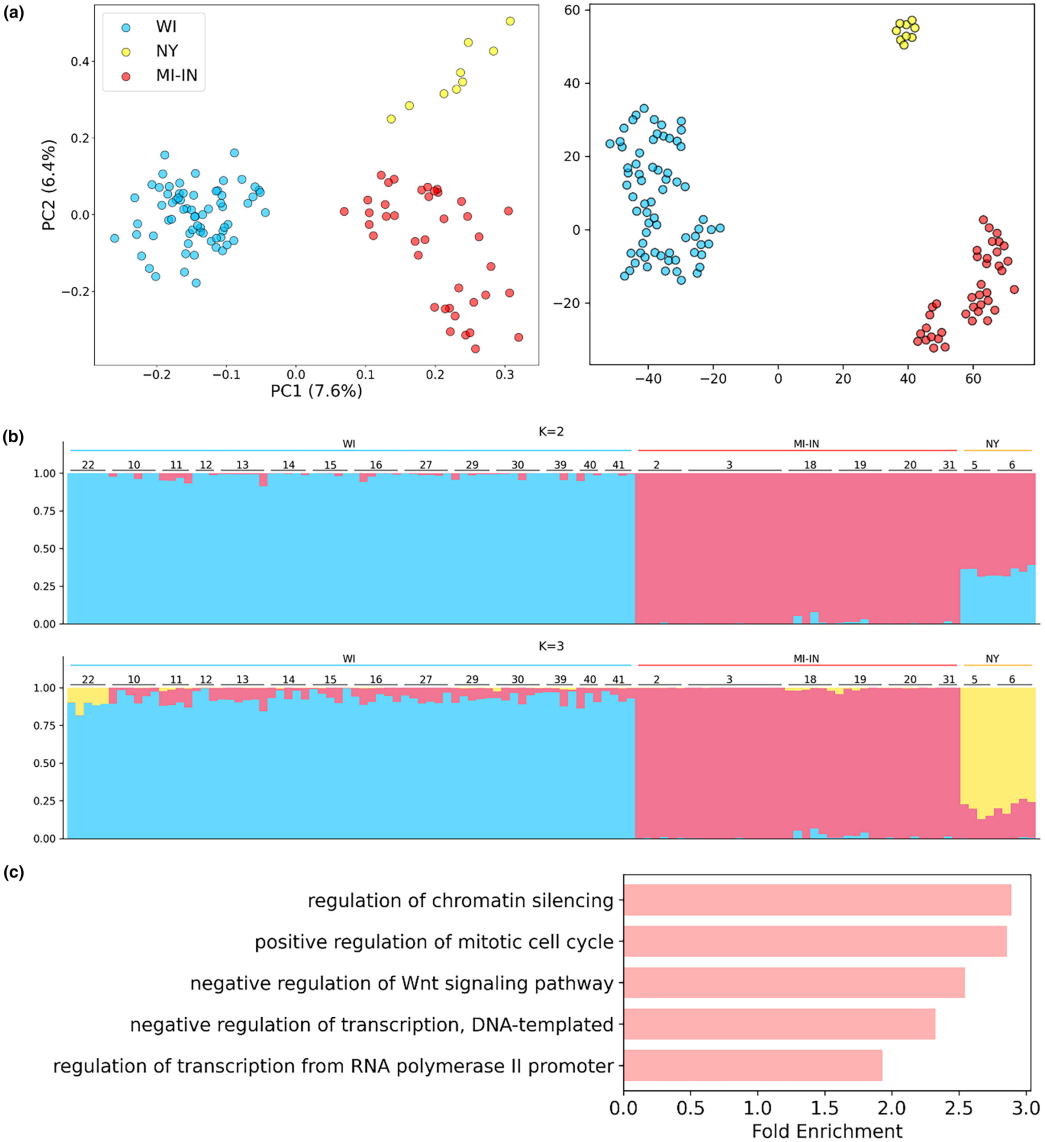
Population structure of *Plebejus samuelis*. (a) The first two principal components (left) and the top 10 components summarized by t‐SNE (right) of *P. samuelis* populations. Each dot in the figure represents one specimen. (b) Population structure by STRUCTURE with preselected population number *K* = 2 and 3. Each bar in the figure represents one specimen, and specimens are labelled by locality. (c) Enriched GO terms associated with proteins show elevated divergence in three major clusters of *P. samuelis*.

We sequenced 3–10 Karner blues from each of 22 localities, resulting in a total of 116 samples with completeness (defined as the ratio of present to missing positions for each sample) above 30% for population analysis. To understand the population structure, we performed principal component analysis. The first two principal components revealed three major clusters, located in Wisconsin, New York, and Michigan–Indiana (Figure 2a left). The separation of Wisconsin from Michigan by Lake Michigan, a biogeographical barrier, may have contributed to the separation of samples from these two neighboring states into different clusters. Additional analyses with t‐SNE revealed all samples still clustering into the three major groups (Figure 2a right), consistent with the results from the first two principal components.

We estimated the likelihood of population numbers (*K*) from 2 to 7 with STRUCTURE. Partitioning into three clusters produced the highest likelihood, which is consistent with the results of our PCA analysis and the geographical distribution of samples (Figure 2b). To understand the genetic mechanism contributing to the divergence of these clusters, we identified the proteins that have diverged among all three clusters (see Section 2). Figure 2c shows the top five enriched GO terms, including transcription regulation and Wnt signaling. These pathways are also frequently diverged in pairs of isolated species and populations due to reproductive or geographical barriers (Cong et al., 2019). We do not expect such divergence to be sufficient to reproductively isolate different Karner blue clusters currently, but prolonged separation of different populations may result in gradual establishment of reproductive barriers due to Dobzhansky–Muller hybrid incompatibilities (Unckless & Orr, 2009).

### 3.3 Investigation of fine‐scale population structure among Karner blue collections

**FIGURE 3:**
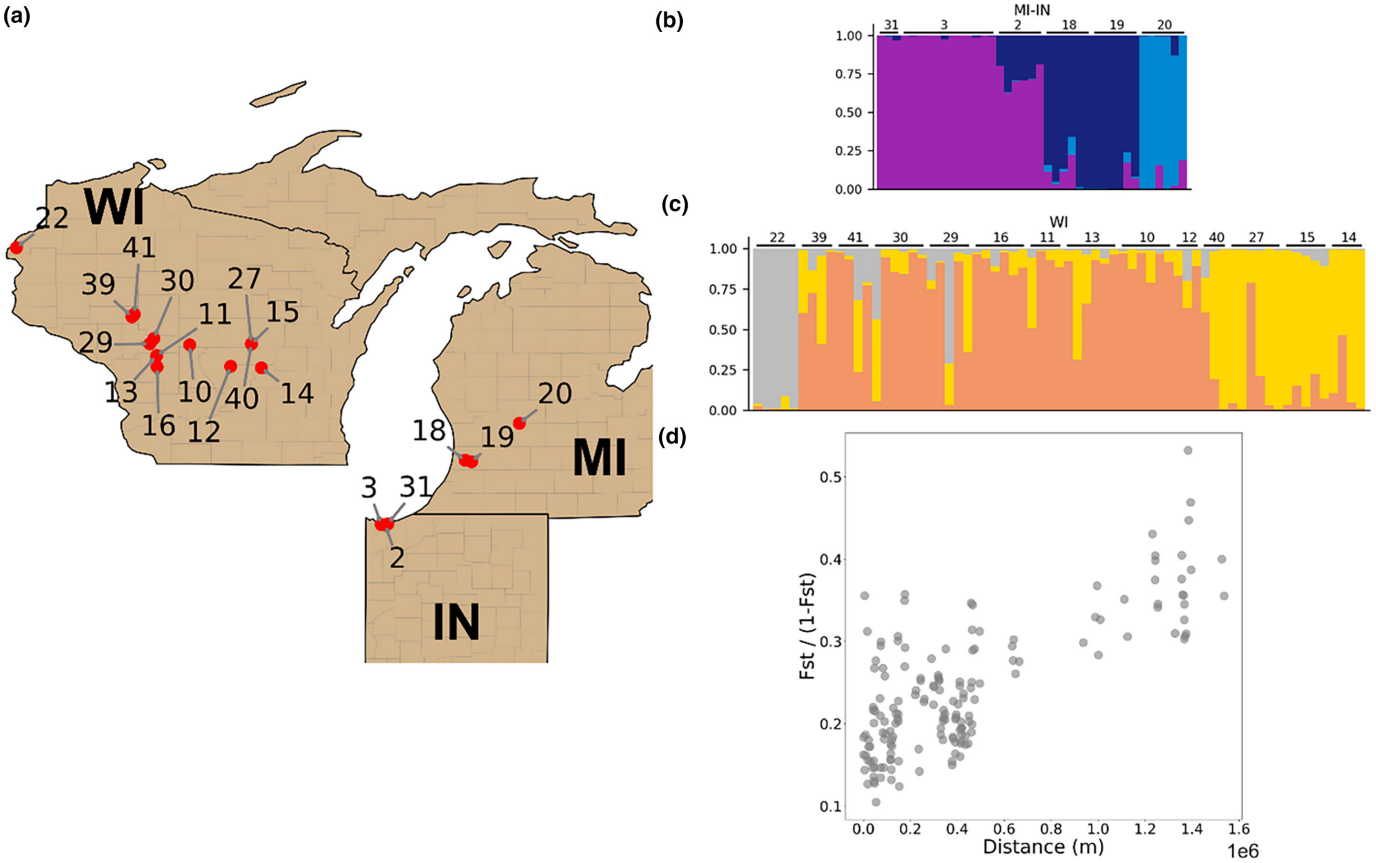
Fine‐scale population structure of *Plebejus samuelis* in Wisconsin and Michigan–Indiana and relationship between genetic diversity and geographical distance. (a) Map of localities from which specimens were collected in WI and MI‐IN. (b) Population structures of specimens collected in MI‐IN, with individuals (bars) grouped by locality. (c) Population structure of specimens collected in WI, with individuals (bars) grouped by locality. (d) Correlation between *F*_ST_/(1 − *F*_ST_) and geographical distance of populations in different localities.

We applied additional hierarchical STRUCTURE analyses using *K* = 2–10 to the Wisconsin and *K* = 2–7 to the Michigan–Indiana clusters, respectively, to evaluate the presence of any additional structuring (Figure 3a). Samples from New York were only collected from two localities, and thus we did not further partition them. For both the Wisconsin and Michigan–Indiana samples, we observed that the likelihood of population structure reached a plateau starting from *K* = 3. The fine‐scale populations among both Michigan–Indiana (Figure 3b) and Wisconsin (Figure 3c) samples mostly agreed with the geographical localities of the samples (Figure 3a), indicating that samples collected from nearby localities share higher genomic similarity. The most northwestern locality sampled, locality 22 in Fish Lake Wildlife Area in northwest Wisconsin, exhibited substantially different population structure compared to other Karner populations (Figure 3c).

The correlation between genomic divergence and geographical distances was also supported by a high correlation (Figure 3d) between *F*_ST_/(1 − *F*_ST_) and geographical distance (Mantel test correlation coefficient *r* = .73, *p* < .01). The higher similarity between samples from nearby localities is expected due to the Karner blue's low dispersal capacity and tendency to remain in a relatively small area and breed locally (Bidwell, 1995; Knutson et al., 1999). For example, Karner blue populations show high inbreeding coefficients ranging from .17 to .45 (Table 1). An inbreeding coefficient above .1 is considered high and may indicate inbreeding depression (Keller & Waller, 2002).

**TABLE 1:** Inbreeding coefficient for each locality sampled.

Locality	Inbreeding coefficient
2	.3983
3	.1700
5	.4223
6	.3685
10	.2181
11	.4523
12	.4434
13	.3336
14	.3167
15	.3278
16	.2971
18	.3677
19	.4026
20	.3515
22	.359
27	.2939
29	.353
30	.3369
31	.4309
39	.3649
40	.4267
41	.3738

*Note*: See Appendix S1 for sample size and other metadata for each locality.

### 3.4 Comparison of Karner blue genomic structure with closely related species

**FIGURE 4:**
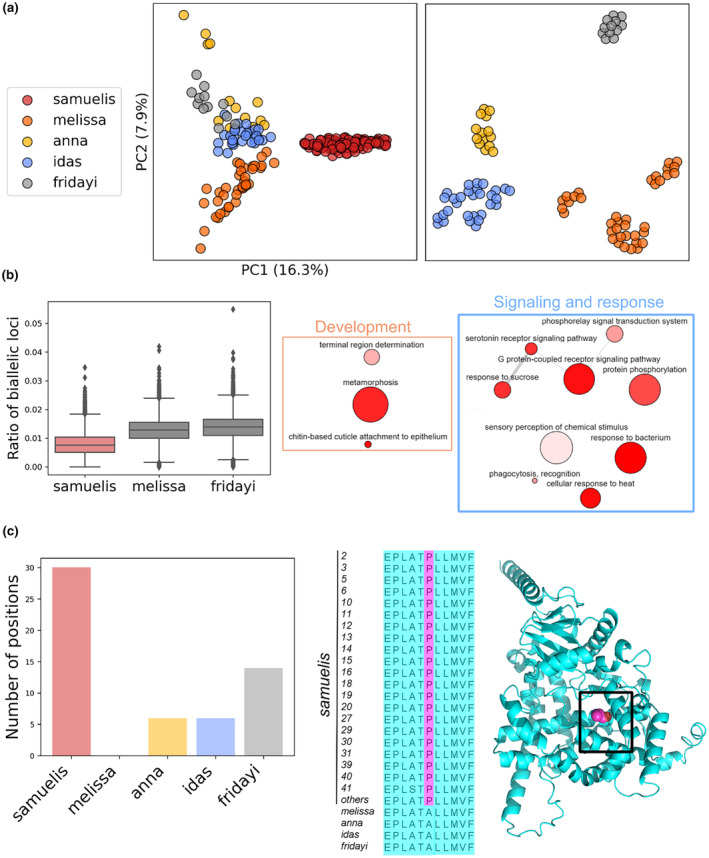
Comparison of *Plebejus samuelis* with other closely related species. (a) The first two principal components of PCA obtained from Z‐linked loci for Karner blues, *Plebejus melissa*, *Plebejus idas*, *Plebejus fridayi*, and *Plebejus anna* (left) and t‐SNE result of the first 9 principal components of PCA on the same dataset excluding Karner blue samples (right). (b) Distribution of the ratio of multi‐allelic loci within non‐overlapping 10‐kb windows in Karner blues, *P. melissa* and *P. fridayi* (left), and enriched GO terms associated with proteins encoded in regions showing “loss of diversity” in Karner blues but elevated divergence in *P. melissa* and *P. fridayi* (middle and right). The diameter of each circle is indicative of the specificity or generality of the associated GO term; more specific GO terms are represented by smaller circles. Additionally, the color intensity of each circle corresponds to the p‐value obtained from enrichment analysis, with darker colors denoting lower *p*‐values. (c) number of unique amino acid variations (left) present in over 95% of *P. samuelis*, *P. melissa*, *P. anna*, *P. idas*, and *P. fridayi*, an alignment (middle) of residue 299–309 of Cytochrome P450 4g1 (ID assigned in our annotation: ENSAPBT00000041574_R1), and illustration of a unique change from Ala to Pro (residue 304) in Karner blues highlighted in magenta (right) and structure of this protein with residue 304 shown as sphere (magenta).

Figure 4a shows the PCA for the Karner blue and its relatives (*P. fridayi*, *P. idas*, and *P. anna*) based on Z‐linked loci. The first two principal components effectively distinguished the Karner blue from other species; however, they did not achieve a clear separation among other species (Figure 4a left). Subsequently, the application of t‐SNE using the first nine principal components further aided in distinguishing these other species (Figure 4a, right). The PCA using nuclear loci also showed similar results (Figure S4). We calculated the ratio of bi‐allelic loci for Karner blue samples in each 10 kb genomic window and compared them to the distribution of such ratios in *P. melissa* and *P. fridayi*. The ratio of bi‐allelic loci in Karner blues was substantially lower than in the other two species: the median ratio was 0.008 for the Karner blues, whereas the medians for *P. melissa* and *P. fridayi* were 0.013 and 0.014 (Figure 4b, left), respectively. Meanwhile, to account for the variation in collection times between Karner blue samples and those of the other two species, we conducted pair‐wise *t*‐tests. These tests compared the ratio of bi‐allelic loci in a pair of Karner blue samples against a pair of *P. melissa* and *P. fridayi* samples. The pairs for comparison were matched such that the geographical distance and the difference in collection years within each pair were at least as great as those in the compared pairs of Karner blues (see Section 2, Appendices S7 and S8). The results of the pair‐wise *t*‐tests revealed a significantly lower proportion of bi‐allelic loci in Karner blue samples in comparison to *P. melissa* (*t*‐statistic: 214, *p* < .01) and *P. fridayi* (*t*‐statistic: 96, *p* < .01). Additionally, we calculate the ratio of bi‐allelic loci for individual samples (Appendix S9) of Karner blue (0.56%) and the other two species (0.7% for *P. melissa* and 0.69% for *P. fridayi*), which should not have biases arising from differences in collection years and geographical distances in the paired comparisons. The *t*‐test (*p* < .01) suggested the bi‐allelic loci are significantly less for individual Karner blue samples compared with the other two species. Thus, the above results suggest the Karner blues have less genetic diversity compared with *P. melissa* and *P. fridayi*. We further identified 10 kb genomic windows with significantly low genetic diversity in the Karner blue but high diversity in *P. melissa* and *P. fridayi*, and we termed these windows as “Karner blue‐specific loss of diversity” regions (see Section 2). GO term enrichment analysis of genes in such regions revealed their involvement in development, chemical signaling, and responses to environmental cues, including heat, sucrose, and bacteria (Figure 4b, middle and right). Therefore, the reduction of genetic diversity in the Karner blue indicates depauperate gene pools on which natural selection can act.

To identify alleles that could potentially impact fitness of the Karner blue, we assumed that the widespread alleles in *P. melissa*, *P. fridayi*, *P. idas*, and *P. anna* are beneficial or at least neutral while rare alleles are more likely to be deleterious. We compared the frequency of amino acids at each protein position in the Karner blue to these closely related species. Our analysis revealed that 30 unique single‐amino acid variations (SAVs) present in over 95% of Karner blue specimens are significantly underrepresented (<5%) in the other species (Appendix S10). However, other species possess much fewer such unique SAVs. *Plebejus melissa*, in particular, does not have a single such unique SAV (Figure 4c left). It is not clear whether these SAVs are phenotypic and a large portion of them might be neutral and do not affect fitness of the Karner blue. However, incorporating structure information may help us prioritize SAVs that are more likely to affect stability or function of proteins. Using structure models predicted by Omegafold, we identified six SAVs that were buried in the core of proteins (relative solvent accessibility <25%). These mutations may potentially diminish protein stability. For example, the residue 304 in Cytochrome P450 4g1 (gene ID in our annotation: ENSAPBT00000041574_R1) is alanine (A) in other species, but it changes to proline (P) in the Karner blue (Figure 4c, middle). The structural model of the protein indicates that this residue is located at the helix in the core of the protein (Figure 4c, right). Although the SAV is unlikely to destroy the protein, it may introduce a kink or bend in the helix, which could affect the protein's structure, stability, and proper function. The closest homolog of Cytochrome P450 4 g1 in *Drosophila* is involved in terminal oxidative decarbonylase in cuticular hydrocarbon biosynthesis within oenocytes, according to Flybase (https://flybase.org/reports/FBgn0010019).

## 4 DISCUSSION

Prior investigations of Karner blue population structure among collections from Michigan, Indiana, and New York (Gompert et al., 2006, 2010) found little evidence of genetic divergence between Karner blues east and west of Lake Michigan. Thus, they suggested that Karner blue conservation and management need not treat geographically separate populations as genetically distinct, but they did encourage further investigation to inform high‐consequence management decisions such as among‐population translocations. Our results from characterizing genome‐wide diversity at a much larger number of loci, however, reveal strong evidence of population structure east and west of Lake Michigan, as well as finer‐scale subdivisions. In addition, our comparisons among the Karner blue and other, more western, *Plebejus* species support the finding of Forister et al. (2011) that the Karner blue is a distinct species.

The high inbreeding coefficients in nearly all populations suggest that inbreeding may have already increased the frequency of deleterious genetic traits. Negative impacts of inbreeding on various butterfly species have been described. Elevated inbreeding in the Glanville fritillary butterfly (*Melitaea cinxia*) was associated with decreased larval survival, egg‐hatching rate, and adult longevity (Saccheri et al., 1998). De Ro et al. (2021) found elevated homozygosity and low estimates of effective population size in the grayling (*Hipparchia semele*) after decades of precipitous declines and low frequency of long‐distance dispersal among fragmented populations. In our study, we attempted to identify candidate deleterious genetic traits by finding alleles that are prevalent in the Karner blue but rare in other closely related species (Figure 4). Some of these alleles could have become fixed due to positive selection and could therefore be beneficial, but some are likely deleterious because of the high inbreeding and low genetic diversity of the Karner blue relative to other abundant *Plebejus* species. Some of these potentially deleterious alleles resulted in SAVs in protein sequences. By incorporating knowledge about protein structure and function, we could further identify the set of unique SAVs that are likely to negatively affect protein stability and/or function. These hypotheses can be further tested through experiments. For example, given the important roles of cuticular hydrocarbons in insect communication, especially in mating and insecticide resistance (Adams et al., 2021; Nojima et al., 2007; Pascale & Thiet, 2016; Snellings et al., 2018), the potential deleterious effect of A304P on Cytochrome P450 4g1 function in the Karner blue may affect communications between Karner blues or their interaction with ants (Pascale & Thiet, 2016), potentially contributing to the decline of some populations' populations. Gene‐editing approaches such as CRISPR may eventually be able to correct deleterious SAVs (Sun et al., 2017). For example, researchers genetically engineered and released sterile mosquitoes to reduce mosquito population sizes and incidence of mosquito‐borne disease (Meghani & Boete, 2018), demonstrating the potential of gene editing to remove disfavored alleles in the populations. However, gene editing of natural populations of any taxon remains rare and controversial due to the risk of potential unintended consequences and public skepticism, although this rarity may change given continuing declines of myriad species (Meek et al., 2023).

Genetic rescue, where low levels of immigration into fragmented and isolated populations are facilitated (e.g., through translocations) to introduce novel genetic variation (Tallmon et al., 2004), has been proposed for different butterfly species but has seen little direct implementation to date. Roitman et al. (2017) proposed translocations into the most urbanized and degraded populations of the Eltham copper butterfly *Paralucia pyrodiscus lucida* based on patterns of historical gene flow and population structure to improve genetic diversity, fitness, and persistence. Similarly, as fragmented populations of the cold‐adapted *Erebia epiphron* continue to contract, Minter et al. (2020) suggested translocations may increase diversity and ability to adapt to future climate change. The Karner blue has already been extirpated from many known former localities and portions of its range including multiple “trailing edge” populations at the warmer and drier periphery of the range, and the loss of additional populations is expected to continue with climate change and ongoing habitat degradation/fragmentation (Hällfors et al., 2016). Despite the high levels of inbreeding measured in our study, genetic differentiation among regions suggests that extant isolated Karner blue populations may still harbor unique genetic diversity and adaptive capacity that could be used to restore population‐level genetic diversity but is at risk of being lost over time due to genetic drift (Hällfors et al., 2016; Thompson et al., 2023). Genetic rescue (Carlson et al., 2014; Souza et al., 2023; Wadgymar et al., 2022) might be one mechanism to facilitate adaptation that could be leveraged for Karner blue conservation, in one of two ways. First, it could be used to increase the resilience and adaptive capacity of existing populations to climate change impacts by restoring any lost beneficial alleles and past levels of genetic diversity that further study of historical specimens might reveal. Second, in the process of creating some new populations in areas beyond the species' historical range that are becoming climatically suitable—a key climate change adaptation strategy that can help this low‐motility species move in concert with movement of the climatic conditions on which it depends (Karasov‐Olson et al., 2021)—it could be employed in the form of intentionally mixing provenances of founding individuals brought together. Additionally, or alternatively, evolutionary rescue (Souza et al., 2023; Wadgymar et al., 2022) could play a role in adaptation of Karner blue populations to changing climate conditions. While genetic rescue relies on increasing genetic diversity by addition of novel genetic variation, evolutionary rescue represents increased frequency of adaptive alleles in a population resulting from selective pressure reducing less adaptive genetic variation (Carlson et al., 2014). The putative ability of a population to reverse population decline through evolutionary rescue would depend on the presence of adaptive genetic variation in a population and the ability to avoid extirpation related to stochastic events. In the case of the Karner blue butterflies in northwestern Indiana within Indiana Dunes National Park (Localities 2, 3, and 31), we saw evidence of low genetic variability and, as documented elsewhere (Patterson et al., 2020), low abundance. Together those suggest limited chance of evolutionary rescue. Under other circumstances—more robust genetic variation and greater abundance—strong selection, such as we observed with historically warm conditions causing phenological mismatching between Karner blue larvae and hostplants, could result in populations better adapted genetically to changing climatic conditions. We do not know if those more favorable conditions for evolutionary rescue might exist in other Karner blue populations but the components for evolutionary rescue—larger, more genetically heterogenous populations—would likely benefit long‐term population survival.

Whole genomic sequencing in this study supports the designation of the Karner blue as a distinct species from the other *Plebejus* species sequenced, reinforcing the conclusions of Forister et al. (2011). *Plebejus melissa* is the closest species geographically to the Karner blue, overlapping slightly on the western edge of the Karner blue range, including Fish Lake Wildlife Area in northwest Wisconsin sampled in this study. Gompert et al. (2006) reported introgression of western *P. melissa* mitochondrial haplotypes into western Karner blues, specifically those in their study sampled from Fish Lake Wildlife Area, but very little evidence of nuclear introgression. Our study was not designed to explicitly evaluate the degree of hybridization between the Karner blue and *P. melissa* at this area of range overlap, but we make a potential contribution toward future studies of hybridization between these species by sequencing the *P. melissa* genome.

The finding of range‐wide and regional Karner blue population structure reveals the species diversity consequences of losing unique populations such as the northern Indiana population, and likely the many other extirpated populations unable to be sampled before their demise. This new understanding can support stewardship of remaining Karner blue populations in several important ways. First, they can help Karner blue conservation focus on safeguarding and expanding small, genetically distinct populations that contribute to the species' overall genetic diversity, such as the geographically disjunct population in and around Fish Lake Wildlife Area in northwest Wisconsin. Second, they can inform contrasting but not necessarily incompatible approaches to conservation translocation. On the one hand, understanding of fine‐scale population structure can support the traditional conservation approach—even in the context of climate change‐motivated conservation translocation—of avoiding mixing independent evolutionary trajectories that potentially possess unique adaptations and adaptive potential. On the other hand, this understanding could inform deliberate genetic mixing—that is, supplementation from distant populations—of some populations to reduce negative impacts of continued genetic isolation by enhancing diversity and perhaps conferring specific adaptations or adaptation potential. Ultimately, effective use of the kind of genomic information provided here to conserve sensitive species like the Karner blue requires thoughtful, forward‐looking, and holistic application (Willi et al., 2022).

## AUTHOR CONTRIBUTIONS

**Jing Zhang:** Conceptualization (equal); data curation (lead); formal analysis (lead); funding acquisition (supporting); investigation (lead); methodology (lead); writing – original draft (lead); writing – review and editing (lead). **Aaron W. Aunins:** Conceptualization (equal); data curation (equal); formal analysis (supporting); funding acquisition (supporting); investigation (lead); methodology (supporting); writing – original draft (equal); writing – review and editing (equal). **Qian Cong:** Conceptualization (equal); data curation (lead); formal analysis (lead); funding acquisition (supporting); investigation (lead); methodology (lead); writing – original draft (lead); writing – review and editing (lead). **Jinhui Shen:** Conceptualization (supporting); data curation (lead); formal analysis (lead); funding acquisition (supporting); investigation (supporting); methodology (lead); writing – original draft (supporting); writing – review and editing (supporting). **Leina Song:** Conceptualization (supporting); data curation (lead); formal analysis (lead); funding acquisition (supporting); investigation (supporting); methodology (lead); writing – original draft (supporting); writing – review and editing (supporting). **Gregor W. Schuurman:** Conceptualization (lead); data curation (lead); formal analysis (supporting); funding acquisition (lead); investigation (supporting); methodology (supporting); writing – original draft (equal); writing – review and editing (equal). **Randy L. Knutson:** Conceptualization (equal); funding acquisition (lead); investigation (equal); writing – original draft (supporting); writing – review and editing (supporting). **Ralph Grundel:** Conceptualization (lead); data curation (lead); formal analysis (supporting); funding acquisition (lead); investigation (supporting); methodology (supporting); writing – original draft (equal); writing – review and editing (equal). **Jessica Hellmann:** Conceptualization (equal); data curation (supporting); formal analysis (supporting); funding acquisition (supporting); investigation (supporting); methodology (supporting); writing – original draft (supporting); writing – review and editing (supporting). **Timothy L. King:** Conceptualization (lead); funding acquisition (lead); investigation (lead). **Nick V. Grishin:** Conceptualization (lead); data curation (lead); formal analysis (lead); funding acquisition (supporting); investigation (lead); methodology (lead); writing – original draft (lead); writing – review and editing (lead).

## CONFLICT OF INTEREST STATEMENT

The authors have no competing interests.

## Supporting information


Figure S1.
Figure S2.
Figure S3.
Figure S4.

Appendix S1.
Appendix S2.
Appendix S3.
Appendix S4.
Appendix S5.
Appendix S6.
Appendix S7.
Appendix S8.
Appendix S9.
Appendix S10.

## Data Availability

The sequence data for all samples are available at NCBI BioProject PRJNA995790, and the *P. melissa* genome assembly is available at NCBI with accession number JBBPCG000000000. Final SNP calls are deposited at U.S. Geological Survey ScienceBase at https://doi.org/10.5066/P143CSPQ.

## Endnote

^1^
Indiana Dunes National Park managers and research partners began augmenting sites in the park with captive‐reared butterflies (from a lab colony descended from Indiana Dunes NP butterflies) in 2011 – after samples for this study were collected – in an attempt to prevent extirpation (Schuurman et al., 2023). These efforts were unsuccessful and the last Karner blue butterfly seen in Indiana Dunes NP was observed in 2014 (Schuurman et al., 2023).
