# Supplementary material for: Range‐wide population genomic structure of the Karner blue butterfly, Plebejus (Lycaeides) samuelis
Source: Ecol Evol. 2024 Sep 12;14(9):e70044. doi: 10.1002/ece3.70044 (PMC11392825; doi:10.1002/ece3.70044)
Supplement: Supplementary file 1 — Figure S1. Figure S2. Figure S3. Figure S4. [file ECE3-14-e70044-s001.pdf]

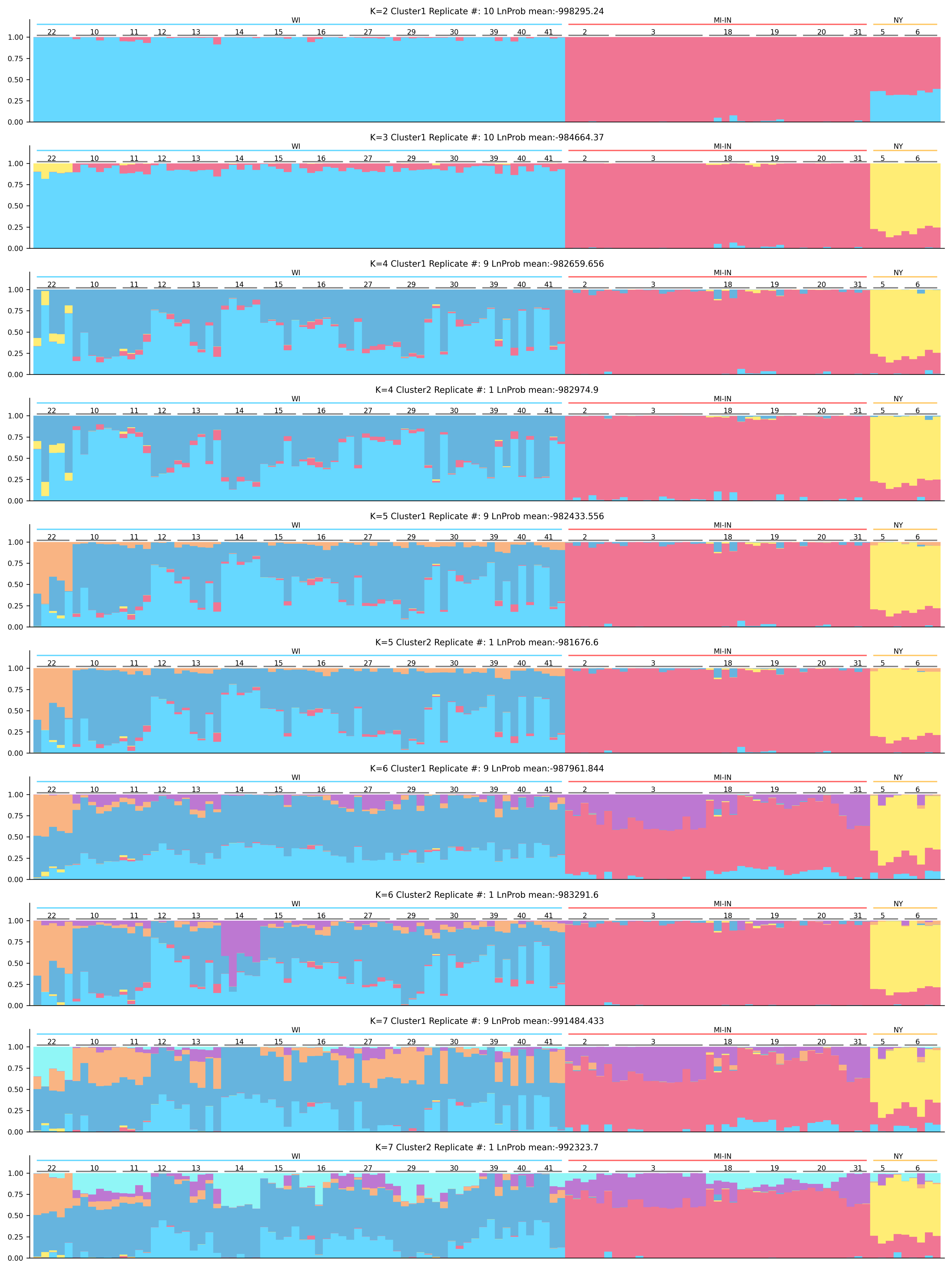

Fig S1. CLUMPAK summary of patterns of STRUCTURE results for all populations. K from 2 to 7 and 10 replicates for each K.

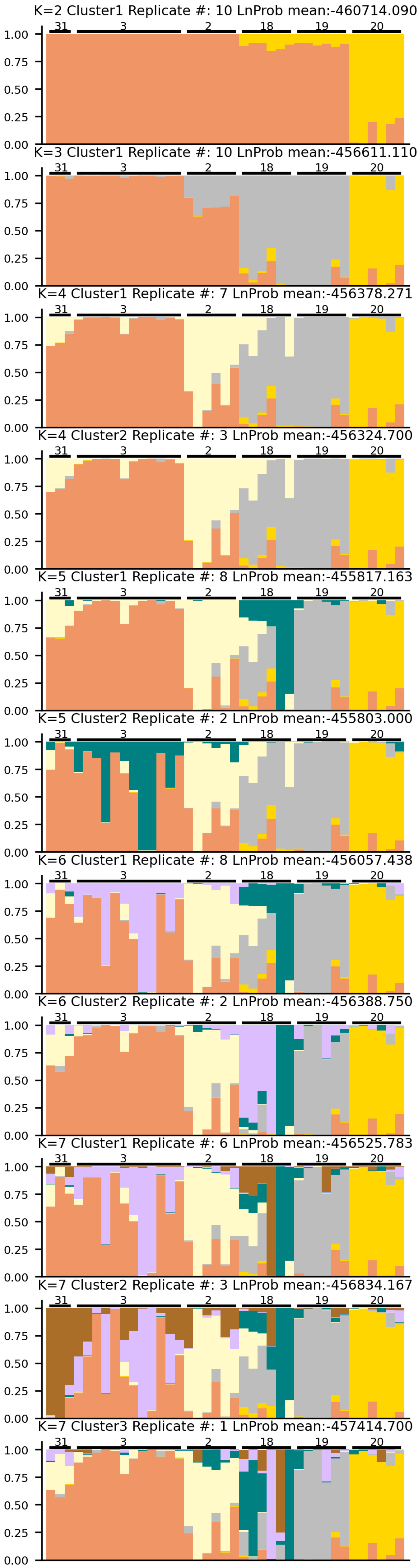

Fig S2. CLUMPAK summary of STRUCTURE patterns for MI and IN populations. K from 2 to 7 and 10 replicates for each K.

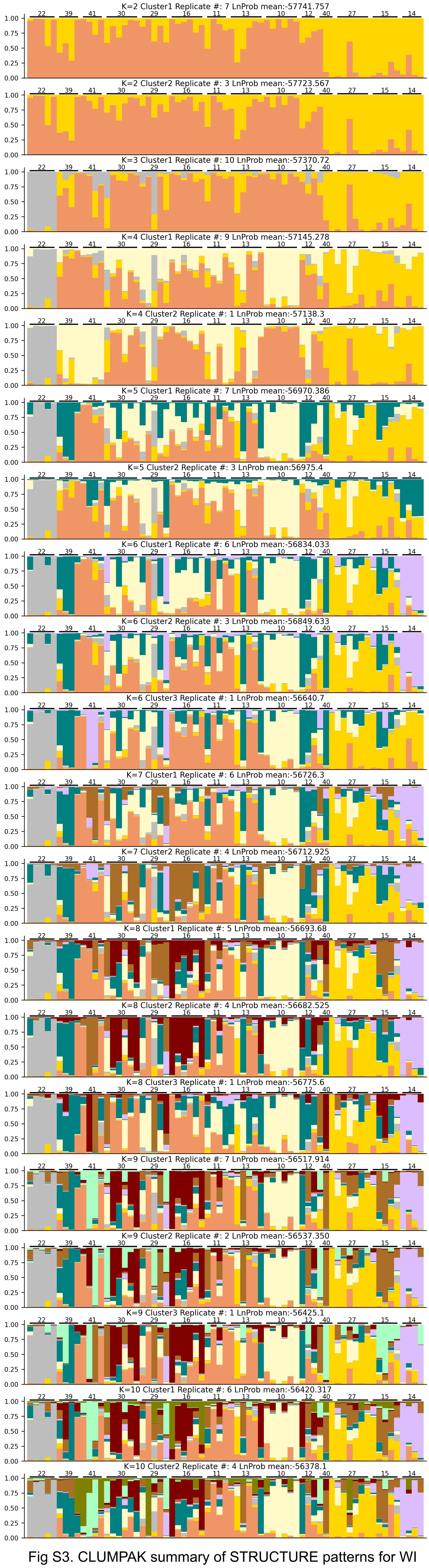

Fig S3. CLUMPAK summary of STRUCTURE patterns for WI populations. K from 2 to 10 and 10 replicates for each K.

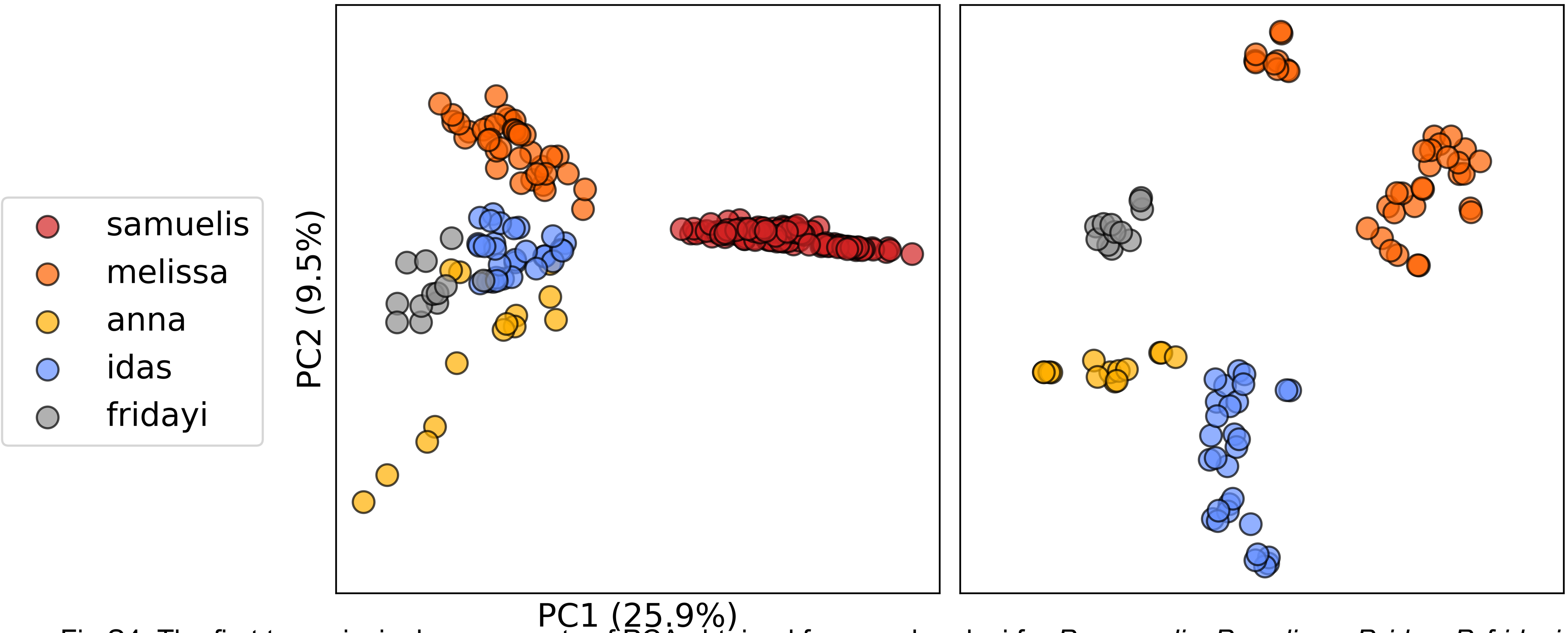

Fig S4. The first two principal components of PCA obtained from nuclear loci for *P. samuelis*, *P. melissa*, *P. idas*, *P. fridayi,* and *P. anna* (left) and t-SNE result of the first 12 principal components of PCA on the same dataset excluding *P. samuelis* samples (right).
